# Supplementary material for: Identification of photoperiod-induced specific miRNAs in the adrenal glands of Sunite sheep (Ovis aries)
Source: Front Vet Sci. 2022 Jul 22;9:888207. doi: 10.3389/fvets.2022.888207 (PMC9354845; doi:10.3389/fvets.2022.888207)
Supplement: Supplementary file 1 [file Data_Sheet_1.ZIP › Supplementary file/miRDeep2 file.zip!/miRDeep2 file/Know_Structure/SP42A2/oar-mir-194.pdf]

miRBase precursor : oar-mir-194
Total read count : 4729
oar-miR-194 read count : 4719
remaining reads : 10

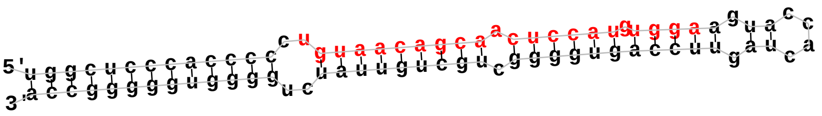

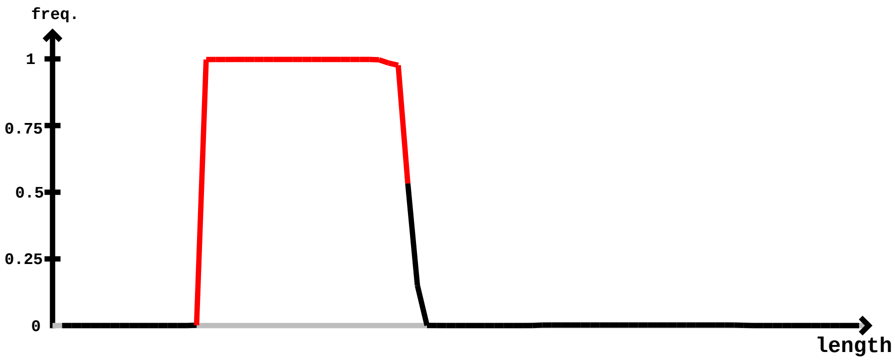

oar-miR-194

```
5'- uggcucccacccccuguaacagcaacuccaugugga aguaccacuaguuccaguggggcugcuguuaucuggggugggggcca   -3'    exp
    ((((((((((((..(((((((((.((((((.(((((.((....)).)))))))))))).)))))))))..))))))))))))    reads    mm    sample
    .............cuguaacagcaacuccaugugg................................................    4        0     seq
    .............cuguaacagcaacuccaugugga...............................................    1        0     seq
    .............cuguaacagcaacuccauguggaa..............................................    1        0     seq
    ..............uguaacagcaacuccaug...................................................    6        0     seq
    ..............uguaacagcaacuccaugu..................................................    58       0     seq
    ..............uguaacagcaacuccaugug.................................................    39       0     seq
    ..............uguaacagcaacuccaugugg................................................    2091     0     seq
    ..............uguaacagcaacuccaugugga...............................................    1808     0     seq
    ..............uguaacagcaacuccauguggaC..............................................    17       1     seq
    ..............uguaacagcaacuccauguggaa..............................................    690      0     seq
    ..............uguaacagcaacuccauguggaag.............................................    1        0     seq
    ..............uguaacagcaacuccauguggaagu............................................    1        0     seq
    ...............guaacagcaacuccaugugg................................................    1        0     seq
    .................aacagcaacuccauguggaa..............................................    1        0     seq
    ...............................................uccaguggggcugcuguuaucu..............    1        0     seq
    ................................................ccaguggggcugcuguuauc...............    1        0     seq
    ................................................ccaguggggcugcuguuaucu..............    4        0     seq
    ................................................ccaguggggcugcuguuaucug.............    3        0     seq
    .................................................caguggggcugcuguuaucug.............    1        0     seq
```
